# TP53 codon 47 and 72 polymorphisms show no association with HPV in Zimbabwean women living with HIV and histologically confirmed cervical and vulvar disease

**DOI:** 10.3389/frph.2026.1808566

**Published:** 2026-04-20

**Authors:** Oppah Kuguyo, Margaret Pascoe, Eugenie Lohmann, Takudzwa Magwaku, Diana L. T. Mukuku, Ardele Mandiriri, Nyarai Soko, Collet Dandara, Rudo Makunike Mutasa, Megan Fitzpatrick, Joel Palefsky, Trine Rounge, Zvavahera Mike Chirenje, Racheal S. Dube Mandishora

**Affiliations:** 1 Division of Human Genetics, Department of Pathology, Faculty of Health Sciences, University of Cape Town, Cape Town, South Africa; 2 Newlands Clinic, Harare, Zimbabwe; 3 Early Detection and Prevention Research, International Agency for Research on Cancer, Lyon, France; 4 Department of Biochemistry and Biotechnology, Faculty of Science, University of Zimbabwe, Harare, Zimbabwe; 5 Medical Microbiology Unit, University of Zimbabwe Faculty of Health Sciences, Harare, Zimbabwe; 6 Department of Histopathology, University of Zimbabwe Faculty of Health Sciences, Harare, Zimbabwe; 7 Department of Pathology, University of Wisconsin, Madison, WI, United States; 8 Department of Medicine, UCSF School of Medicine, San Francisco, CA, United States; 9 Department of Research, Cancer Registry of Norw,aY, Oslo, Norway; 10 Department of Obstetrics and Gynaecology, University of Zimbabwe Faculty of Health Sciences, Harare, Zimbabwe

**Keywords:** HIV, HPV, HIV opportunistic infections, cervical cancer, vulvar cancer, Sub-Saharan Africa, CIN, VIN

## Abstract

**Introduction:**

Interaction between HIV and human papillomavirus (HPV) increases the risk of HPV persistence. However, a few studies have explored the interplay between host genetic factors and HPV in women living with HIV (WLWH). We report on a study investigating the role of TP53 mutations among HPV DNA-positive cervical and vulvar tissue from Zimbabwean WLWH.

**Methods:**

This cross-sectional study recruited 102 WLWH aged over 18 years, with histologically confirmed vulvar (*n* = 13) or cervical disease (*n* = 89). Vulvar or cervical tissue biopsies, preserved in formalin-fixed paraffin-embedded (FFPE) blocks, were retrieved. Tumour DNA was extracted and analyzed for 17 HPV genotypes using the ATILA multiplex system. The DNA was also examined for TP53 variants located in codon 72 and 47 using Sanger sequencing. Associations between HPV genotypes and TP53 variants were assessed using logistic regression.

**Results:**

The histological classifications had the following proportions: cervical intraepithelial neoplasia (CIN)1: 10%, CIN2: 4.5%, CIN3: 82%, and cervical cancer: 3.4%. Vulvar disease was classified as: vulvar intraepithelial neoplasia (VIN)3: 84.6%, and vulvar cancer (15.4%). HPV16 prevalence was 40% and 64% in vulvar tissue. Codon 72G and codon 47 T alleles had frequencies of 34.1% and 2.9% in cervical tissue, and 29.2% and 4.2% in vulvar tissue, respectively. There was no significant association between TP53 codon 72 and 47 mutations, and HPV genotypes (*p* > 0.05).

**Discussion and Conclusion:**

These findings underscore the need to conduct further research to identify other genetic variants within the TP53 gene that may contribute to the role of TP53 in HPV susceptibility that has been previously reported in other studies.

## Introduction

Human papillomavirus (HPV) commonly infects the reproductive tract of sexually active individuals (1). The persistence of some human papillomavirus genotypes in the cervix and anogenital regions leads to the development of cervical and a subset of anogenital cancers including vulvar, anal and vaginal (2). HPV genotypes, that are classified as high-risk, account for >95% of cervical cancers, and up to 75% of vulvar cancers (3, 4). Both cervical and vulvar cancers greatly threaten the health of women living in sub-Saharan Africa (SSA), leading to death if not diagnosed and treated early (5).

Only less than 1% of HPV-infected individuals develop HPV-related cancers in their lifetime, indicating the need for investigating factors involved in carcinogenesis. A primary example is the Human Immunodeficiency Virus (HIV), an established driver of HPV persistent infections, that significantly increases the risk of carcinogenesis (6–8). Women living with HIV (WLHIV) are at a 4-fold and 6-fold higher risk of acquiring HPV and developing HPV-related cancers, respectively, compared to HIV-uninfected women (9, 10). Furthermore, infection with hrHPVs increases the risk of HIV acquisition, on account of the highly inflammatory vaginal environment that is induced by HPV positivity (11).

In the pathogenesis of high-risk HPV, oncoproteins E6 and E7 target tumour suppressor proteins such as *TP53* and pRb, respectively, to dysregulate the normal protein activity. Of great interest to this study is *TP53*, which is key to cell cycle regulation by repairing DNA damage, initiating apoptosis, and modulating cell proliferation, all of which, when deregulated, are hallmarks of cancer (12). Therefore, variations in the *TP53* genome sequence can be useful biomarkers for hrHPV persistence and cervical cancer.

The highly conserved DNA binding domain of *TP53*, exons 5–8, has been frequently described as the hotspot for mutations within the gene. The most widely researched is the *TP53* codon 72 single nucleotide polymorphism (SNP), which results in the substitution of the amino acid proline with arginine in a proline-rich region (13). The *TP53* codon 72 G allele translates to a less stable p53 isoform that is more susceptible to degradation, compared to the wild type (C allele). The *TP53* codon 72 G is associated with higher apoptosis, increased DNA repair mechanisms and an overall decreased genomic instability – potentiating the decreased risk of carcinogenesis (14). However, there has been conflicting data on the role of *TP53* codon 72 in hrHPV infection, persistence and cervical carcinogenesis in different populations and sub-populations. As a result, the relationship between *TP53* codon 72 and HPV or cervical cancer is still unknown, prompting the need for further research in diverse populations.

Moreover, there is limited data on the role of other genetic variants within the conserved region of *TP53,* such as at the codon 47 position. The exact functional role of the *TP53* codon 47 is not well understood; however, this variant has been reported to be prevalent in African populations and has shown the potential of modulating the functionality of the p53 protein. The dearth of available data coupled with the evidence of genetic diversity of African populations calls to attention the need to describe genetic factors of HPV among African populations where HIV is endemic and there are limited diagnostic and screening tools.

The present study aims to determine the frequency of genetic variants in *TP53* (codon 72 and 47) in Zimbabwean WLWH with cervical and vulvar disease, and determine their association with HPV positivity. This study builds on previous data where genetic variants were associated with HPV positivity, however, no association between *TP53* codon 72 and HPV was reported (15). In the present study, we employ robust genetic characterisation methods to detect *TP53* genetic variants among WLWH, and investigate the understudied codon 47 variant which has been detected mostly among African populations. This study is of significance because WLWH in Zimbabwe have higher HPV prevalence (16) higher prevalence of oncogenic HPV infection (17), and a more diverse HPV profile compared to HIV negative women (18). Determining genetic variants associated with HPV is an important step to identifying biomarkers that can be used to screen WLWH that are more prone to HPV related cancers, to administer preventive measures, towards better health outcomes.

## Materials and methods

### Study design and setting

This cross-sectional study recruited participants from Newlands Clinic, an HIV care and treatment centre located in Harare, Zimbabwe. Participants’ biospecimens were retrieved from Lancet Pathologies, CIMAS diagnostics and Baines private laboratories, and were sectioned at the University of Zimbabwe Faculty of Medicine and Health Sciences, Histopathology unit. Biospecimen analyses were done at the University of Cape Town, Human Genetics Division, in South Africa.

### Ethical approval and informed consent

All study procedures were in accordance with the Helsinki Declaration of 2013. Ethical approvals were obtained from the Medical Research Council of Zimbabwe (MRCZ/A/2396) and the Research Council of Zimbabwe (Permit number: 03879). All participants were administered information to participate verbally by the research assistant and provided the informed consent form to read through. We obtained consent to participate in writing. Consent was administered in English or Shona (the main native language spoken in the city spoken in Harare).

### Inclusion/exclusion criteria

Women who were aged at least 18 years, sexually active, HIV-1 positive, and reporting for HIV antiretroviral therapy and management of opportunistic infections were eligible for recruitment into this study. To be included in the study, women had to have histologically confirmed diagnoses of cervical and/or vulvar pre-cancer and cancer lesions, with respective biopsies collected through Newlands Clinic between 2016 and 2019. Individuals failing to meet these criteria and being unable to give written informed consent were excluded from the study.

### Study participants

At enrolment, the participant's data such as age, histological diagnosis, and disease stage were collected from their clinical records. To maintain confidentiality, participant data were deidentified, and all participants were assigned a unique identifier, that was used to associate their data to the respective biospecimen throughout the study.

### Biospecimen retrieval

Archived and deidentified formalin-fixed paraffin-embedded (FFPE) blocks were retrieved and sectioned to 3–4 µm scrolls that were placed into two separate cryotubes tubes assigned with the respective unique identifiers. Histology slides were prepared at the beginning and end of sectioning each block, and a Histopathologist from Parirenyatwa hospital confirmed that the sectioned scrolls had tumour tissue using the haematoxylin and eosin stain.

### Genomic DNA extraction and quality check

Genomic DNA was extracted from each cryotube with FFPE scrolls, using a modified protocol from Zymo genomic DNA extraction kit for FFPE, utilizing a slightly modified protocol (Zymo Research, California, USA). Briefly, a xylene-based deparaffinization solution was used to remove the paraffin layer, and the tissue was digested for 12 h using 10 mg/mL Proteinase K (Zymo Research, California, USA). The DNA was bound to a Spin Column, washed in 70% ethanol, and eluted in DNA/RNA-free water. Quantity and quality of DNA were checked using a Nanodrop^TM^ Spectrophotometer ND-1000 (Nanodrop^TM^, ThermoFisher, Denver, USA). Absorbances at 230 nm, 260 nm and 280 nm were used to compute the purity of the extracted DNA. Only DNA that had absorbance ratios for 230/260 and 260/280 ratios of 1.8–2.0 were included in this study for further analysis.

### HPV genotyping

DNA was used to genotype 17 HPVs, using the ATILA Genotyping Fluorescent High-risk HPV Detection Kit (ATILA Biosystems, USA), as described in the manufacturer's protocol. Only samples with a positive amplification of the internal beta-globin control (IC+) were considered for further analysis.

### Characterisation of *TP53* codons 47 and 72

Genomic DNA was characterised for the loci of interest using Sanger Sequencing. Amplification of *TP53* exon 4 was done using the International Agency Research on Cancer (IARC) designed primers (IARC protocol, 2019): 5’-TGCTCTTTTCACCCATCTAC-3’ (forward) and 5’- ATACGGCCAGGCATTGAAGT-3’ (reverse primer) (Inqaba Biotechnical Industries, Pretoria, South Africa). A 25*μ*l PCR reaction mix with 1 ng/µL template DNA, 5X Green Go Taq Reaction Buffer (Promega Cooperation, Madison, USA), 5 mM dNTPs (Kappa Biosystems, Cape Town, South Africa), 5U/µL Go Taq Polymerase (Promega Illinois, USA), 25 mM MgCl_2_ (Thermo Scientific, Waltham, USA), 100 µM forward primer, 100 µM reverse primer was run in an Applied Biosystems SimpliAmp^TM^ Thermal Cycler (Thermo Scientific, USA). Thermocycling conditions were initial denaturation at 95°C for 3 min followed by 35 cycles of denaturation at 95°C for 30 s; annealing at 55.7°C for 30 s; and extension at 72°C for 1 min; and a final extension at 72°C for 5 min. Unbound PCR reagents were removed from the PCR templates using FastAp™ Thermosensitive Alkaline Phosphatase (*FastAP*^TM^) and Exonuclease I (*ExoI*^TM^) (Thermo Scientific, Waltham, USA). The Big-Dye Terminator V3.1 cycle sequencing kit (Life Technologies, CA, USA) was used for sequencing following the manufacturer's protocol. The ethanol/EDTA precipitation was used for the template purification, and highly de-ionized formamide was used to denature DNA in preparation for electrokinetic injection in the ABI 3130 Genetic Analyzer capillary (ThermoFisher Scientific, New York, USA), using the POP-7^TM^ polymer with 80 cm capillary length protocol.

### Data analyses

STATA/BE 17.0 (StataCorp LLC, Station College, TX, USA) was used to analyse data. Demographic and HPV prevalence data were assigned into continuous or categorical data. Continuous data were expressed into mean ± standard deviation while categorical data were expressed into absolute/relative frequencies, respectively. Unpaired T-tests and Wilcoxon's unparametric T-tests were applied to compare continuous and categorical data. Chromas Pro Ver 2.0 (Technelysium, Australia) was used to visualise, assemble, and analyse *TP53* Exon 4 chromatograms. Adherence of the SNPs to Hardy Weinberg equilibrium was tested using the Pearson's chi-square test. Linkage disequilibrium and haplotype analysis were evaluated using SHEsis. SHEsis was also used to calculate genotypic and allelic frequencies of the two SNPs being investigated. GraphPad Prism^TM^ version 8.0.2 (GraphPad^TM^ Software Inc., San Diego, California, USA) was used for bivariate analysis where the association between allelic variants and phenotypes for two groups was done using the unpaired non-parametric t-test and for more than two groups was done using One-way ANOVA T-test. A value of *P* < 0.05 was considered to indicate a statistically significant result. Statistical power analysis of the study was done using *post-hoc* Power Calculator from ClinCalc (ClinCalc LLC, Illinois, USA). Descriptive analysis and correlation matrix with Pearson coefficient was performed using the Jamovi statistical program version 2.2. Ggplot2 3.3.3 treemap 2.4–2 and ComplexUpset were used for graphics under R software 4.1.0. The R function (glm) was used to calculate the logistic regression (family = binomial (link = “logit”) controlling for age. rs1800371 (CC, CT), rs1042522 (CC, CG, GG) have been tested against each infection group [High-risk, Low-risk, Quadrivalent vaccine (6/11/16/18), 16/18, Mono-infections, Multiple infections, HPV 16, HPV 18, HPV 31, HPV 33, HPV 35, HPV 39, HPV 45, HPV 51, HPV 52, HPV 53, HPV 56, HPV 58, HPV 59, HPV 66, HPV 68, HPV 6, HPV 11]. Adjusted odds ratios, 95% confidence intervals and *p*-values were used to determine significance.

## Results

This cohort was made up of 102 WLWH, 89 (87%) with cervical disease (pre-cancerous + cancerous) and 13 (13%) with vulval disease (precancerous + cancerous). The overall mean age for the cohort was 43 years (SD = 9), with an age range of 25 to 64 years (Table 1). The distribution of cervical disease was cervical intraepithelial neoplasia (CIN) 1 (10.1%), CIN2 (4.5%), CIN3 (82%) and invasive cervical cancer (3.4%). All participants with vulval disease either presented with VIN3 (84.6%) or cancer (15.4%). Two (2%) participants had both CIN3 and vulval cancer. Age and histopathological stage were not associated with cervical or vulval disease.

**Table 1:** Demographic characteristics of participants in the study (*n* = 102).

Characteristic	Cervical (%) (*n* = 89)	Vulvar (%) (*n* = 13)	Dual site (%) (*n* = 2)	*p* -value ^a^
Age:				
0–30 years	8 (9.0)	0 (0.0)		
31–40 years	28 (31.5)	8 (61.5)	2 (100.0)	
41–50 years	34 (38.2)	4 (30.8)		
51–60 years	16 (18.0)	1 (7.7)		
61–70 years	3 (3.3)	0 (0.0)		
Mean ± SD	43 ± 9 years	39 ± 7 years		0 . 081
Disease stage:				
Cervical:				
CIN1	9 (10.1)	–		
CIN2	4 (4.5)	–		
CIN3	73 (82.0)	-–	2 (100.0)	
Cervical cancer	3 (3.4)			
Vulvar:				
VIN 3	–	11 (84.6)	–	
Vulval cancer	–	2 (15.4)	2 (100.0)	

CIN, cervical intraepithelial neoplasia; VIN, vulval intraepithelial neoplasia. Total sample size: HPV genotyping were performed for the 102 WLWH enrolled in this study. Of these, 100 biospecimens were successfully genotyped, and two cervical tissue were excluded from further analysis because, there was failure to detect the beta-globin internal control. Therefore, this study is comprised of cervical tissue *n* = 87; vulvar tissue *n* = 13.

^a^ Comparison between cervical and vulval disease Dual-site cases refer to participants with lesions identified at more than one anatomical site.

**Figure 1:**
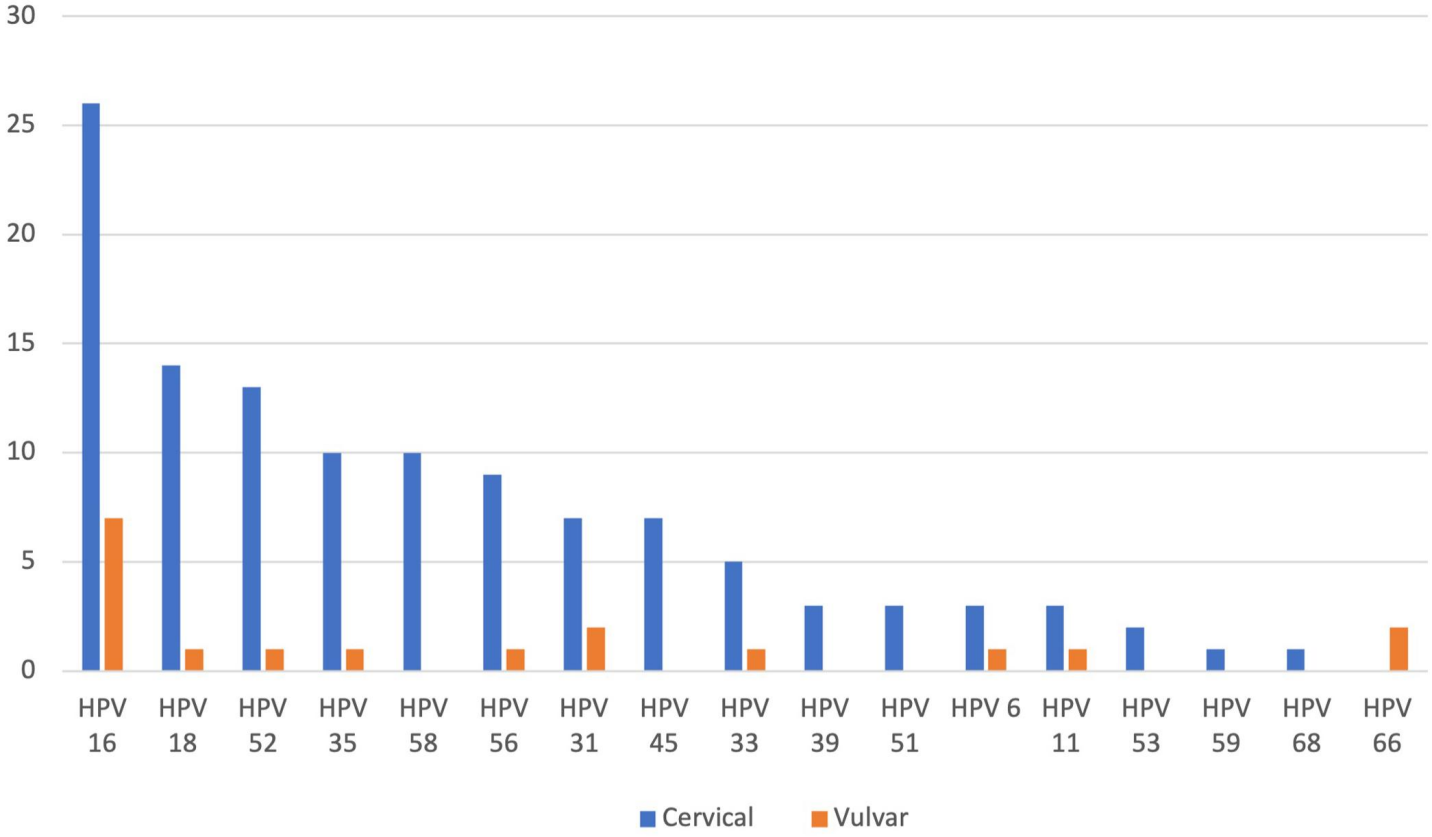
Distribution of HPV genotypes among women living with HIV that presented with cervical (*n* = 65) and vulvar (*n* = 11) precancerous and cancerous lesions.

HPV genotyping were performed for the 102 WLWH enrolled in this study. Of these, 100 biospecimens were successfully genotyped, and two cervical tissue were excluded from further analysis because, there was failure to detect the beta-globin internal control. Therefore, this study is comprised of cervical tissue *n* = 87; vulvar tissue *n* = 13. Distribution of HPV genotypes by location is summarised in Figure 1. The overall prevalence of HPV DNA was 75% in cervical tissue and 85% in vulvar tissue. HPV 16 was the most common genotype detected in both cervical tissue (40%; *n* = 26/65) and vulvar tissue (64%; *n* = 7/11). Other commonly observed genotypes in cervical tissue included HPV 18 (22%), HPV 52 (20%), HP V35 (15%) and HPV 58 (15%). In vulvar tissue HPV 31 (2%), HPV 66 (2%), HPV 33 (1%), HPV 6 (1%) and HPV 11 (1%) were detected. From the 3 WLWH presenting with confirmed cervical cancer, multiple infections with HPVs 16, 45 and 58 were detected. While the 2 patients with vulvar cancer presented with multiple infection with HPVs 16, 18, 33, 35, and 52. Eight HPV types were detected only in cervical tissue, high-risk HPV types: 58, 45, 39, 51, 53, 59, 68 and low-risk type: HPV 6. HPV 66 was exclusive to two vulvar samples. Twenty-two samples were positive for HPV consensus DNA but were not positive for any of the specific genotypes included in the assay.

Forty WLWH (40%) presented with multiple HPV genotypes (Table 2). The highest number of HPV co-infections observed in 2 cervical tissues was 5.

**Table 2:** Number of HPV infections detected in cervical and vulvar samples (*n* = 100).

Number of HPV types	Cervical (%) (*n* = 87)	Vulvar (%) (*n* = 13)
0	22 (25.3)	2 (15.3)
1	31 (35.6)	5 (38.5)
2	23 (26.4)	5 (38.5)
3	6 (6.9)	1 (7.7)
4	3 (3.5)	0 (0)
5	2 (2.3)	0 (0)

Total sample size: HPV genotyping were performed for the 102 WLWH enrolled in this study. Of these, 100 biospecimens were successfully genotyped, and two cervical tissue were excluded from further analysis because, there was failure to detect the beta-globin internal control. Therefore, this study is comprised of cervical tissue *n* = 87; vulvar tissue *n* = 13.

**Figure 2:**
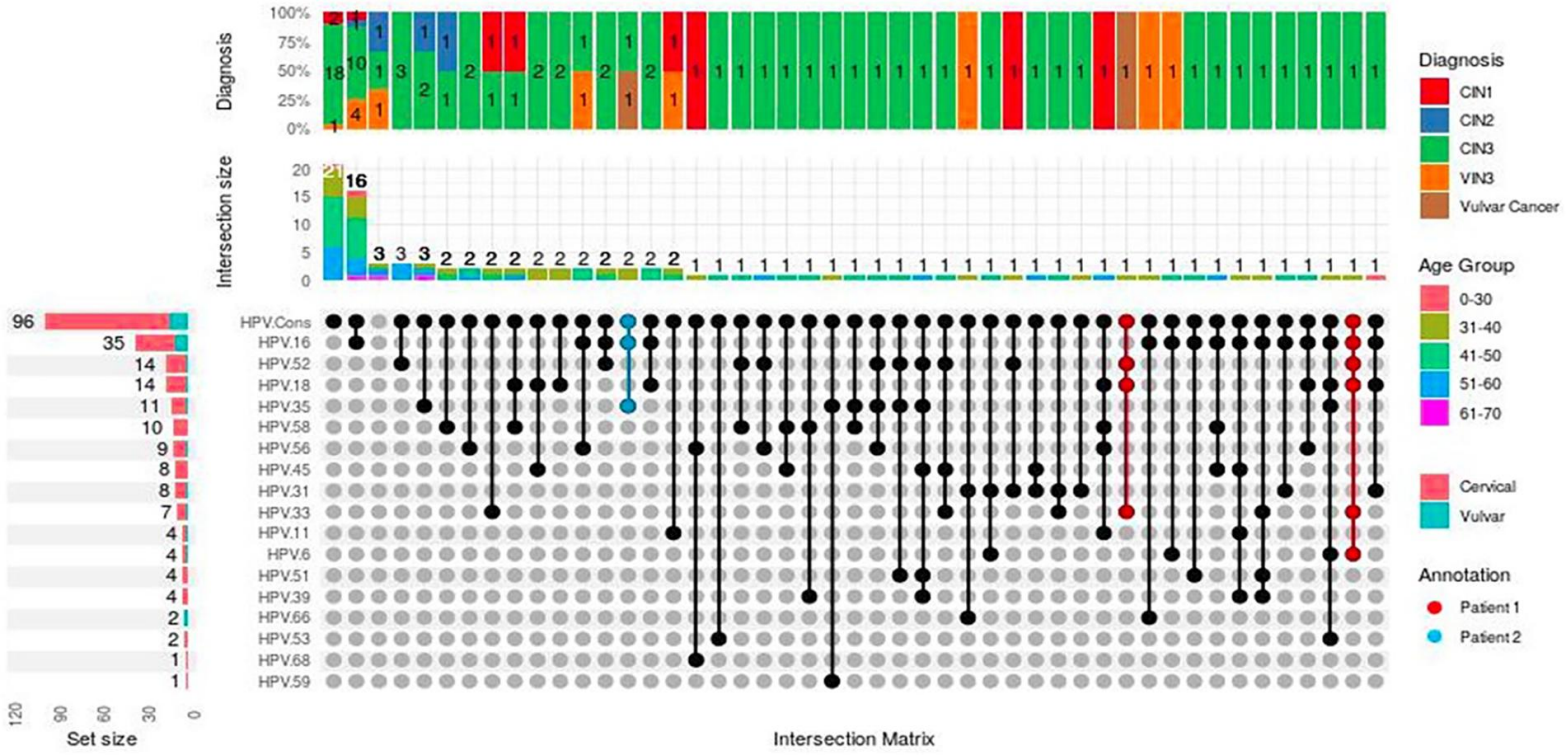
Upset plot of genotype distribution by age group and diagnosis. This figure illustrates the intersection of genotyping profiles for 102 samples analyzed across 17 different HPV targets. The identified genotypes are included in the intersection matrix. Each row of this matrix is dedicated to a specific HPV type, with ‘HPV detected’ indicating positive HPV detection in 100 of the samples. The vertical columns represent a total of 46 genotype combinations. The bar plot on the left displays the number of cases with frequencies in cervical or vulvar regions. The frequency bar plot for each genotype is color-coded by age group. Additionally, a stacked bar plot presents the distribution of diagnoses, with the number of cases indicated within each subgroup.

The distribution of HPV infections stratified by age and diagnoses were computed (Figure 2). Sixteen percent (*n* = 16) of WLWH had HPV 16 mono-infections, while 3% (*n* = 3) had HPV 35 and 52 mono-infections, and 2% (*n* = 2) were infected with HPV 18. HPVs 16 (17%, *n* = 17), 18 (12%, *n* = 12), and 52 (9%, *n* = 9) were the most common multiple infections detected in both cervical samples. Analyses of the profiles for the 2 WLWH with both vulvar cancer and CIN3 showed the presence of HPV genotypes 18, 33, and 52 DNA in tissue from both vulvar and cervix’ woman 1’ and HPVs 16 and 35 DNA positivity in both sites of ‘woman 2’. Both participants with CIN3 and vulvar cancer were aged between 31 and 40 years old. The two WLWH infected by HPV 66 were under the 31–40 years age category and also diagnosed with VIN3. Four percent (*n* = 4) WLWH were positive for HPV 11 DNA (*n* = 3 cervical, *n* = 1 vulvar). Among these cases, two women had a single HPV 11 infection and were aged between 31 and 50 years old and either diagnosed CIN1 or VIN3. The distribution of distinct HPV genotypes were comparable by age (*p* > 0.05), except for HPV 18 (*p* < 0.05) Supplementary Figure 1.

**Figure 3:**
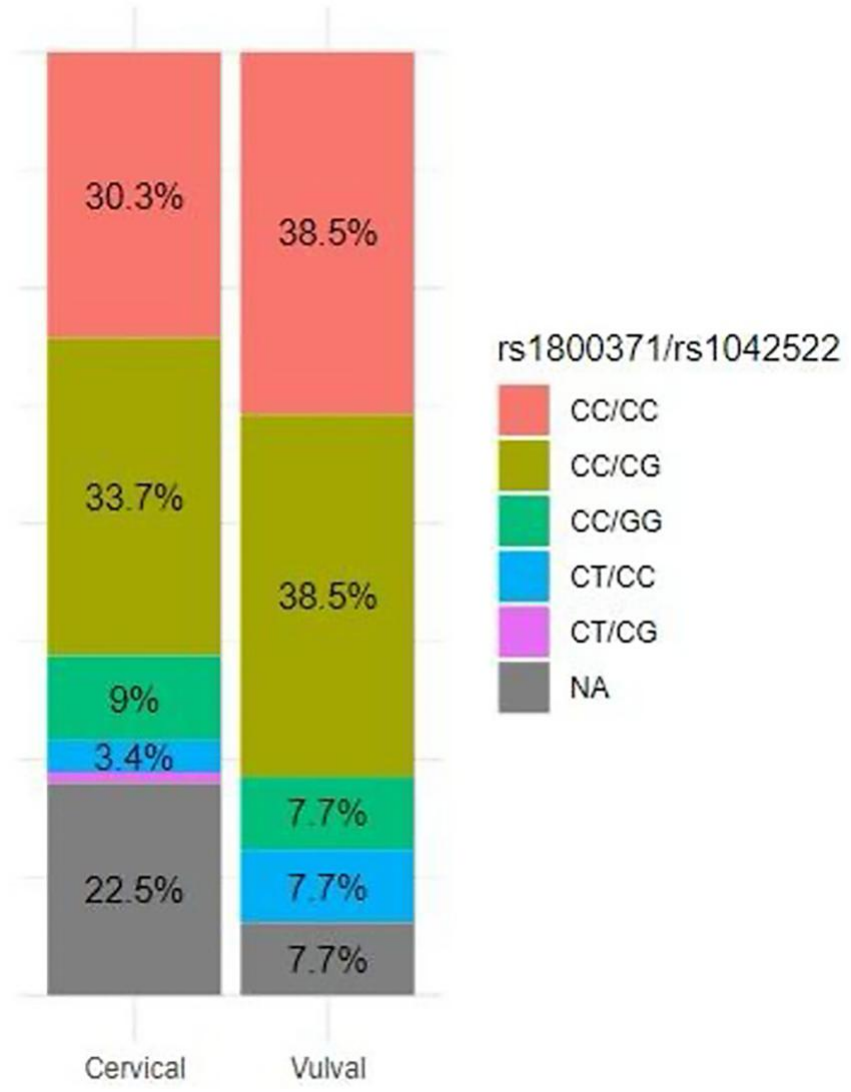
Stacked bar chart showing the percentage distribution of rs1800371/rs1042522 variant combinations in cervical tissue (69 samples) and vulvar tissue (12 samples) with the relative frequencies of each combination.

*TP53* tumour tissue mutation characterization was successfully performed for 69 cervical and 12 vulvar tissue samples. The remaining samples (*n* = 21) were excluded for failure to amplify. The distribution of the rs1800371 (codon 47) and rs1042522 (codon 72) haplotype variants in cervical and vulval tissue showed that the CC/CG haplotype was most common in cervical (33.7%) and vulval tissue (38.5%) (Figure 3). The variant allele frequencies (VAFs) for codon 47 and codon 72 were comparable in both cervical (*p* = 0.700) and vulval tissue (*p* = 0.446). Both SNPs were in Hardy-Weinberg Equilibrium (HWE) in both cervical and vulval tissue (Table 3).

**Table 3:** Allelic and genotype frequencies of TP53 exon 4 codon 47 (rs1800371) and codon 72 (rs1042522) for cervical and vulvar samples (*n* = 81).

Disease	N	SNP	Variant allele frequency	Genotype frequency			HWE *p*-value
Cervical	69	Codon 47	**T**	**CC**	**CT**	**TT**	
		(rs1800371)	0 . 029	0 . 942	0 . 058	**0**	0 . 804
	69	Codon 72	**G**	**CC**	**CG**	**GG**	
		(rs1042522)	0 . 341	0 . 435	0 . 449	0 . 116	0 . 998
Vulvar	12	Codon 47	**T**	**CC**	**CT**	**TT**	
		(rs1800371)	0 . 042	0 . 917	0 . 083	**0**	0 . 880
	12	Codon 72	**G**	**CC**	**CG**	**GG**	
		(rs1042522)	0 . 292	0 . 500	0 . 417	0 . 083	0 . 977

Univariate regression analyses showed that none of the genetic polymorphisms in *TP53* were associated with vulval disease (Supplementary Table S1). Heterozygote carriers of *TP53* codon 47 (rs1800371 CT) were protected against type specific HPV DNA positivity, namely HPV 16 (OR = 0.1; 95% CI = 0.0–0.4; *p* = 0.001), HPV18 (OR = 0.1; OR = 0.0–0.6; *p* = 0.017), HPV52 (OR = 0.1; 95% CI = 0.0–0.9; *p* = 0.037) in univariate regression. While *TP53* codon 72 (rs1042522) GG was a protective factor against HPV 16 (OR = 0.1; 95% CI = 0.0–0.7; *p* = 0.01), HPV 18 (OR = 0.1, 95% CI = 0.0- 0.7, *p* = 0.022) and HPV 52 (OR =  0.1; 95% CI = 0.0–0.9; *p* = 0.037) in cervical tissue in univariate regression. However, in multivariate regression analyses adjusting for age, histological diagnosis and number of HPVs (Table 4), none of the *TP53* genetic polymorphisms were associated with any of the HPV genotypes (*p* > 0.05).

**Table 4:** Multivariate regression analyses associating the distinct HPV genotypes with **TP53** host genetic polymorphisms.

Predictor/Covariate	aOR (95% CI)	*P* -value
HPV 16 (*R*^2^ = 0.2182)		0.0037(global)
Age	1.0 (0.9–1.1)	0.658
Histological Diagnosis	NA	NA
Number of HPV types	2.6 (1.4–5.1)	0.05
*Tp53* codon 47	1.6 (0.1–19.4)	0.715
*Tp53* codon 72	0.4 (0.0–22.0)	0.321
HPV 18 (*R*^2^ = 0.3101)		0.001(global)
Age	0.9 (0.8–1.1)	0.534
Histological Diagnosis	0.9 (0.3–2.2)	0.753
Number of HPV types	3.2 (1.5–6.8)	0.003*
*Tp53* codon 47	1.2 (0.03–46.1)	0.905
*Tp53* codon 72	1.0 (0.3–3.3)	0.997
HPV 31 (*R*^2^ = 0.1827)		0.48 (global)
Age	1.0 (0.8–1.1)	0.71
Histological Diagnosis	2.2 (0.08–62.3)	0.65
Number of HPVs	1.7 (0.6–4.4)	0.28
*Tp53* codon 47	14.8 (0.6–339.4)	0.09
*Tp53* codon 72	1.7 (0.2–11.8)	0.59
HPV 33 (*R*^2^ = 0.3024)		0.06 (global)
Age	1.0 (0.9–1.2)	0.63
Histological Diagnosis	0.5 (0.1–2.0)	0.31
Number of HPV	3.1 (1.3–7.5)	0.01*
*Tp53* codon 47	-	-
*Tp53* codon 72	2.1 (0.3–12.7)	0.43
HPV 35 (*R*^2^ = 0.0737)		0.52 (global)
Age	1.0 (0.9–1.1)	0.69
Histological Diagnosis	1.1 (0.3–4.0)	0.80
Number of HPVs	1.7 (0.9–3.1)	0.09
*Tp53* codon 47	-	-
*Tp53* codon 72	0.0 (0.0–2.9)	0.11
HPV 39 (*R*^2^ = 0.3747)		0.06 (global)
Age	1.1 (0.9–1.3)	0.22
Histological Diagnosis	2.0 (0.2–23.4)	0.59
Number of HPVs	4.5 (1.2–16.7)	0.03*
*Tp53* codon 47	-	NA
*Tp53* codon 72	5.2 (0.6–47.2)	0.14
HPV 45 (r^2^ = 0.3301)		0.02 (global)
Age	1.0 (0.9–1.2)	0.95
Histological Diagnosis	17.1 (0.7–426.2)	0.08
Number of HPVs	2.8 (1.2–6.6)	0.02*
*TP53* codon 47	-	-
*TP53* codon 72	4.0 (0.7–23.1)	0.13
HPV 51 (*R*^2^ = 0.2056)		0.40 (global)
Age	1.1 (0.9–1.3)	0.44
Histological Diagnosis	1.6 (0.2–12.9)	0.64
Number of HPVs	2.8 (1.2–6.6)	0.22
*Tp53* codon 47	-	-
*Tp53* codon 72	-	-
HPV 52 (*R*^2^ = 0.127)		0.21 (global)
Age	1.0 (0.9–1.3)	0.72
Histological Diagnosis	0.9 (0.3–2.7)	0.89
Number of HPV types	1.8 (1.0–3.0)	0.03*
*Tp53* codon 47	1.6 (0.1–20.1)	0.71
*Tp53* codon 72	0.5 (0.1–1.7)	0.28
HPV 53 (*R*^2^ = 0.2073)		0.45 (global)
Age	1.0 (0.9–1.2)	0.71
Histological Diagnosis	1.8 (0.1–47.6)	0.72
Number of HPVs	2.6 (0.8–8.3)	0.11
*Tp53* codon 47	-	-
*Tp53* codon 72	2.8 (0.3–25.3)	0.18
HPV 56 (*R*^2^ = 0.2182)		0.45 (global)
Age	1.1 (1.0–1.2)	0.09
Histological Diagnosis	0.6 (0.2–2.4)	0.48
Number of HPVs	1.7 (0.9–3.3)	0.11
*Tp53* codon 47	2.8 (0.2–36.7)	0.42
*Tp53* codon 72	0.4 (0.0–1.5)	0.25
HPV 58 (*R*^2^ = 0.0865)		0.35 (global)
Age	1.0 (0.9–1.1)	0.76
Histological Diagnosis	0.6 (0.2–1.5)	0.25
Number of HPVs	1.7 (1.0–2.9)	0.07
*Tp53* codon 47	-	-
*Tp53* codon 72	1.4 (0.5–4.2)	0.55
HPV 59 (*R*^2^ = 0.3124)		0.52 (global)
Age	0.7 (0.3–1.4)	0.28
Histological Diagnosis	14.2 (0.0 – 50.2)	0.52
Number of HPVs	0.7 (0.1–4.1)	0.68
*Tp53* codon 47	-	-
*Tp53* codon 72	6.2 (0.1–386.5)	0.39
HPV 6 (*R*^2^ = 0.0669)		0.95 (global)
Age	1.0 (0.8–1.3)	0.69
Histological Diagnosis	1.7 (0.0–111.3)	0.81
Number of HPV types	1.6 (0.3–7.3)	0.52
*Tp53* codon 47	-	-
*Tp53* codon 72	2.2 (0.1–37.3)	0.58
HPV 11 (*R*^2^ = 0.5719)		0.01 (global)
Age	1.0 (0.9–1.3)	0.65
Histological Diagnosis	0.1 (0.0–0.8)	0.04*
Number of HPV types	5.2 (1.0–26.3)	0.04*
*Tp53* codon 47	-	-
*Tp53* codon 72	728.1 (0.5–123.7)	0.13

## Discussion

The primary aim of this study was to investigate the HPV genotype-specific distribution in cervical and vulvar tissue in a group of Zimbabwean women, attending a HIV clinic for monitoring. Furthermore, we investigated the association of *TP53* codon 47 and codon 72 polymorphisms with these HPV genotypes detected. To our knowledge, this is one of the first studies from Zimbabwe to explore both viral distribution and host genetic profiles in cervical and vulvar cancer. Building on our previous work examining *GSTP, GSTT1, XRCC1*, and *CASP8* genetic variations in cervical cancer cases using blood samples (15), the current study advances the field by focusing on pre-malignant lesions (CIN1 to CIN3) using FFPE tissue samples. This approach offers novel insights into the genetic underpinnings of HPV-related lesions. We suggest that future research should further investigate sample-related differences in polymorphic analysis to refine our understanding of these associations. HPV genotype profiles for cervical tissue have been widely reported in Zimbabwe for both WLWH and HIV-uninfected women. In the present study, HPVs 16 and 18 were the most reported, corresponding with the published data in Zimbabwe (16–20). Moreover, a high prevalence of genotypes such as HPV 35 (10%), HPV 56 (9%), HPV 39 (3%), HPV 51 (3%) and HPV 53 (2%) was also observed in cervical tissue, in this cohort, even though these HPV genotypes are not incorporated in any of the available HPV prophylactic vaccines. The presence of HPV 35 in WLWH warrants further analyses, given the recent association of this genotype with cervical cancers in different geographic regions (21).

HPV has been associated with the development of a small subset of vulvar lesions and cancer. HPV positivity in the vulvar has been associated with poor prognosis and disease recurrence (22). Even with the high prevalence of HPV in Zimbabwe (17%–27%), especially in WLHIV (33%), there have not many studies analyzing the prevalence of HPV in vulvar tissue in Zimbabwe (19). In this study, 75% and 85% of cervical and vulvar biopsies were positive for HPV DNA respectively. The selected HPV genotyping method inherently reports negative when samples do not have any of the 17 genotypes it assays. It is possible that some samples were positive for HPV DNA but not the genotypes picked by assay and hence HPV positivity can be expected to be higher than 25.3%. Validation for false negatives may have assisted in accurately identifying biospecimens with HPV DNA. In this study, HPV 16 (7%) was the most prevalent genotype in vulvar tissue, consistent with the published literature (23, 24). HPVs 31 (2%), 66 (2%), 11 (1%), 6 (1%), 33 (1%), 56 (1%), 35(1%), 52 (1%) and 18 (1%) were also detected, suggesting that these genotypes may also play a role in the onset or progression of vulvar disease in agreement with previous data (23–25). HPVs 66, 56, and 35 are not encompassed in any available HPV vaccines, potentiating the need to develop broader and inclusive HPV vaccines that can provide adequate protection. Although we are among the first to report on HPV genotypes in vulvar tissue within a Zimbabwean cohort, the prevalence of distinct HPV genotypes observed in this study may not fully represent the burden of HPV in vulvar disease in Zimbabwe, due to the small sample size of vulvar tissue analyzed. Further research is essential to understand better the extent of HPV in vulvar disease, particularly in under-represented and vulnerable populations such as Moreover, the prevalence of the distinct HPV genotypes in vulvar tissue recorded presently may not accurately represent the burden of HPV in vulvar disease in Zimbabwe, on account of the small sample size of vulvar tissue analysed in this study.

Evidence that individuals vaccinated against specific HPV genotypes can be protected against a host of HPV genotypes via immunological cross-protection and cross-reaction has been provided (26). Factors such as HPV phylogenetic diversity, immune suppression, and duration since vaccination also affect cross-protection (27). In our data, the diverse HPV genotypes detected, may potentiate limited protection provided by the vaccines in heterogenous populations such as the topical cohort. Moreover, in Zimbabwe the HPV vaccination program began in 2018, administering the bivalent vaccine that targets HPV 16/18 and there has been conflicted data on the duration of protection elicited by this bivalent vaccine obtained from other countries (28–30). With the evidence of the diverse HPV genotype profile observed in Zimbabwe, it may be of greater benefit to consider administering the more diverse nanovalent HPV vaccine. Taken together, these data point to the essentiality of developing an African-specific HPV vaccine to ensure maximal coverage of HPV genotypes that are prevalent in Africa, protection of these populations who are burdened with HIV and are at a much greater risk of HPV acquisition and persistence compared to other populations.

This study also described the association of two genetic polymorphisms in the tumour suppressor gene, *TP53*, (codon 72 and 47) with distinctive HPV genotypes in cervical and vulvar tissue. The *TP53* codon 72 has been widely studied in relation to HPV and related diseases, and, in our study population, there was no association between *TP53* codon 72 G with any of the HPVs. Our findings are consistent with a previous study from Zimbabwe (31), and some data obtained from African and Asian populations (22, 32, 33). Contrastingly, data from Bangladesh found TP53 C to be associated with cervical cancer risk (34). However, in TP53 codon 72 G has been associated with cervical precancerous lesion, and cancer among Tunisian (35).

Although we were unable to compare frequency of the variant with normal tissue, the frequency of *TP53* codon 72 polymorphism in our precancerous lesions did not differ much from frequencies in healthy African individuals as previously reported elsewhere and in public databases like dbSNP (36). Interestingly, enough data obtained from populations of European ancestry has been largely conflicted, with some studies reporting a lack of association in agreement with our study, while other contradicting studies have found a strong association between *TP53* codon 72 G and HPV persistence or cervical cancer (37, 38). The antithetic relationship between *TP53* codon 72 and HPV/cervical cancer can be attributed to differences in the frequency of the G observed in different population groups (39). In this study, the average G allele frequency was 32%, which is significantly lower than the frequencies reported in European (78%) and Asian (60%) ancestry populations (35). Genetic interactions with environmental factors such as distance from the equator, latitude and ecological adaptation can lead to allelic variation in different ethnic groups (39, 40). Therefore, in some populations, variants such as the *TP53* codon 72 G may have significant functionality in some populations, but not others. Therefore, variants such as *TP53* codon 72 may not be useful as universal HPV biomarkers but are instead population-specific.

The data presented here also reports on a 2.9% prevalence of *TP53* codon 47 T allele in cervical tissue, and 4.2% in vulvar tissue, resonating with other African population data (41–43). The codon 47 variant is described as “African-centric” for its presence among African populations (up to 8%), which is higher than what has been observed in African Americans (1.2%), Finnish Europeans (0%) and Caucasian Americans (0%) (41–43). We found no association between *TP53* at codon 47 (rs1800371) and HPV reported in this study, resonating with data conducted on Bangladeshi women (44). Further studies are needed to identify genetic variants in *TP53* that may increase the role of this gene in cancer susceptibility and prognosis.

## Study limitations

One limitation of this study was that we analysed samples that were available, potentially limiting the power of the study. In future, we will conduct effective sample size calculation to ensure sufficient study power. As a result, we interrogated a small sample size, particularly for vulvar tissue, which resulted in wide confidence intervals in the association analyses for vulvar data. Future studies with larger sample sizes are needed to explore potential biomarkers for HPV-related vulvar disease better. Our study did not evaluate or control for important factors such as viral load, CD4/8 counts, and BMI in the statistical analyses due to missing data. These factors may influence the risk of *TP53* mutations or polymorphisms, as well as the relationship between HPV and cervical/vulvar cancer. The predominance of pre-malignant lesions (93%) in the study cohort may have limited the detection of certain associations, this focus on early-stage disease provides valuable insight into the genetic factors associated with the progression of HPV-related lesions. Future research will broaden the range of lesion stages and incorporate a more comprehensive set of variables to further strengthen the findings.

Our study focused only on HIV infected women and lacked use of normal tissue comparisons of the *TP53* polymorphisms. These limitations impede differential comparison of TP53 polymorphism frequencies between HIV infected women and HIV uninfected women with precancerous lesions thus limiting accurate differential inference between the two distinct groups and the ability to decipher any effects HIV may have on *TP53* during the process of carcinogenesis. In addition, comparisons of normal vs. precancerous lesions allows understanding of any genetic changes that occur as a result of infection. Future studies should look at differential mutations and gene expression according to HIV status.

Our study was also limited because we conducted a cross-sectional study where we recruited participants from a single site, an HIV referral centre – potentially introducing selection bias. Future research should look into a prospective study that recruits participants from multiple recruitment sites to reduce risk of selection bias. In addition, functional studies should also be conducted in to determine the functional significance of the variants. Another limitation of this study is the absence of an HPV-positive, lesion-free comparator group, which limits assessment of variant-specific persistence and progression risk; this will be addressed prospectively in future longitudinal studies.

Emerging evidence also points to the role of vaginal microbiota in driving HPV persistence and cancer development (45). Our study was limited in that we did not consider the microbiome in our analyses. Future studies, should incorporate vaginal microbiota analyses, towards obtaining a holistic understanding in variability in disease risk, and advancing personalised medicine.

## Conclusion

Genotypes such as HPV 35, 39, 51, 53 and 56 were detected in cervical and vulvar tissue in Zimbabwean WLWH, underscoring the heterogeneity of HPV in Africa. The data presented here underscore the need for broader vaccines that include prevalent genotypes in Africa. This study also reports on the relationship between two genetic polymorphisms in *TP53* codons 72 and 47, and HPV in women living with HIV in Zimbabwe. Although no direct relationship was observed between *TP53* codons 47 and 72 and HPV in the current cohort, the current data confirm the presence of the *TP53* codon 47 T allele, which has been shown to be a strong prognostic marker for chemotherapy. Future studies to analyse the role of the *TP53* codon 47 T allele in treatment response among African cancer patients can be conducted, towards personalising therapy.

## Data Availability

The datasets presented in this study can be found in online repositories. The names of the repository/repositories and accession number(s) can be found below: https://www.ncbi.nlm.nih.gov/dbvar/, SUB15751431.

## References

[1] Brianti P De Flammineis E Mercuri SR. Review of HPV-related diseases and cancers. New Microbiol. (2007) 40(2):80–5. 28368072

[2] de Martel C Georges D Bray F Ferlay J Clifford GM. Global burden of cancer attributable to infections in 2018: a worldwide incidence analysis. Lancet Glob Health. (2020) 8(2):180–90. 10.1016/S2214-109X(19)30488-7 31862245

[3] Rakislova N Saco A Sierra A Del Pino M Ordi J. Role of human papillomavirus in vulvar cancer. Adv Anat Pathol. (2017) 24(4):201–14. 10.1097/PAP.0000000000000155 28590952

[4] World Health Organisation. Cervical cancer (2022). Available online at: https://www.who.int/news-room/fact-sheets/detail/cervical-cancer (Accessed March 27, 2023).

[5] MacDuffie E Sakamuri S Luckett R Wang Q Bvochara-Nsingo M Monare B Vulvar cancer in Botswana in women with and without HIV infection: patterns of treatment and survival outcomes. Int J Gynaecol Cancer. (2021) 31(10):1328–34. 10.1136/ijgc-2021-002728 PMC8675890 34493586

[6] Dube-Mandishora RS Christiansen IK Chin’ombe N Duri K Ngara B Rounge TB Genotypic diversity of anogenital human papillomavirus in women attending cervical cancer screening in Harare, Zimbabwe. J Med Virol. (2017) 89:1671–7. 10.1002/jmv.24825 28390142

[7] Isaguliants M Bayurova E Advoshina D Kondrashova A Chiodi F Palefsky JM. Oncogenic effects of HIV-1 proteins, mechanisms behind. Cancer (Basel). (2021) 13(2):305. 10.3390/cancers13020305 PMC7830613 33467638

[8] Stelzle D Tanaka LF Lee KK Khalil IA Baussano I Shah ASV Estimates of the global burden of cervical cancer associated with HIV. Lancet Glob Health. (2020) 9(2):161–69. 10.1016/S2214-109X(20)30459-9 PMC7815633 33212031

[9] Moodley JR Hoffman M Carrara H Allan BR Cooper DD Rosenburg L HIV and pre-neoplastic and neoplastic lesions of the cervix in South Africa: a case-control study. BMC cancer. (2006) 6:135. 10.1186/1471-2407-6-135 16719902 PMC1481580

[10] Zayats R Murooka TT McKinnon LR. HPV and the risk of HIV acquisition in women. Front Cell Infect Microbiol. (2022) 12:814948. 10.3389/fcimb.2022.814948 35223546 PMC8867608

[11] Leibenberg LJP McKinnon LR Yende Zuma N Garrett N Baxter C Kharsany ABM. HPV infection and the genital cytokine milieu in women at high risk of HIV acquisition. Nat Commun. (2019) 10(1):5227. 10.1038/s41467-019-13089-2 31745084 PMC6863918

[12] Hanahan D. Hallmarks of cancer: new dimensions. Cancer Discov. (2022) 12(1):31–46. 10.1158/2159-8290.CD-21-1059 35022204

[13] Khan MA Tiwari D Dongre A Sadaf, Mustafa S Das CR Exploring the p53 connection of cervical cancer pathogenesis involving north-east Indian patients. PLoS One. (2020) 15(9):e0238500. 10.1371/journal.pone.0238500 32976537 PMC7518589

[14] Shen CC Cheng WY Lee CH Dai XJ Chiao MT Liang YJ Both p53 codon 72 arg/arg and pro/arg genotypes in glioblastoma multiforme are associated with a better prognosis in bevacizumab treatment. BMC Cancer. (2020) 20(1):709. 10.1186/s12885-020-07210-8 32727419 PMC7391574

[15] Kuguyo O Dube-Mandishora RS Soko N Magwaku T Matimba A Dandara C. GSTP, GSTT1, XRCC1 and CASP8 genetic variations are associated with human papillomavirus in women with cervical cancer from Zimbabwe. Future Virol. (2024) 19(1):19–32. 10.2217/fvl-2023-0154

[16] Zia N Dube-Mandishora RS Chirenje Z Makunike-Mutasa R Munjoma M Gomo E. Prevalence and genotype distribution of high risk human papillomavirus among HIV infected and uninfected women in Harare, Zimbabwe. Cent Afr J Med. (2023) 69(1-6):1–13.

[17] Kufa T Mandiriri A Shamu T Dube-Mandishora RS Pascoe MJ. Prevalence of cervical high-risk human papillomavirus among Zimbabwean women living with HIV. South Afr J HIV Med. (2024) 25(1):1–7. 10.4102/sajhivmed.v25i1.1633 PMC12242646 40642514

[18] Kuguyo O Dube-Mandishora RS Thomford NE Makunike-Mutasa R Nhachi CF Matimba A High-risk HPV genotypes in Zimbabwean women with cervical cancer: comparative analyses between HIV-negative and HIV-positive women. PLoS One. (2021) 16(9):e0257324. 10.1371/journal.pone.0257324 34582476 PMC8478215

[19] Baay MF Kjetland EF Ndhlovu PD Deschoolmeester V Mduluza T Gomo E Human papillomavirus in a rural community in Zimbabwe: the impact of HIV co-infection on HPV genotype distribution. J Med Virol. (2004) 73(3):481–5. 10.1002/jmv.20115 15170646

[20] Manyere NR Dube Mandishora RS Magwali T Mtisi F Mataruka K Mtede B Human papillomavirus genotype distribution in genital warts among women in Harare, Zimbabwe. J Obstet Gynaecol. (2019) 40(6):830–6. 10.1080/01443615.2019.1673710 31790323

[21] Pinheiro M Gage JC Clifford GM Demarco M Cheung LC Chen Z Association of HPV35 with cervical carcinogenesis among women of African ancestry: evidence of viral host interaction with implications for disease intervention. Int J Cancer. (2020) 147(10):2677–86. 10.1002/ijc.33033 32363580 PMC11090644

[22] Butt JL Botha MH. Vulvar cancer is not a disease of the elderly: treatment and outcome at a tertiary referral centre in South Africa. SAMJ. (2017) 47(11). 10.7196/samj.2017.v107i11.12497 29262943

[23] Halec G Alemany L Quiros B Clavero O Hofler D Alejo M Biological relevance of human papillomaviruses in vulvar cancer. Mod Pathol. (2017) 30(4):549–62. 10.1038/modpathol.2016.197 28059099

[24] Preti M Rotondo JC Holzinger D Micheletti L Gallio N McKay-Chopin S Role of human papillomavirus infection in the aetiology of vulvar cancer in Italian women. Infect Agents Cancer. (2020) 15:2020. 10.1186/s13027-020-00286-8 PMC7110671 32266002

[25] Sznurkowsi JJ Zawrocki A Biernat W. The overexpression of p16 is not a surrogate marker for high-risk human papillomavirus genotypes and predicts clinical outcomes for vulvar cancer. BMC cancer. (2016) 16(1):465. 10.1186/s12885-016-2503-y 27411473 PMC4944532

[26] Pruski D Laggiedo-Zelazowska M Millert-Kalinska S Sikora J Jach R Przybylski M. Immunity after HPV vaccination in patients after sexual initiation. Vaccines (Basel). (2022) 10(5):728. 10.3390/vaccines10050728 35632487 PMC9144159

[27] Stanley M Joura E Yen GP Kothari S Luxembourg A Saah A Systematic literature review of neutralizing antibody immune responses to non-vaccine targeted high-risk HPV types induced by the bivalent and the quadrivalent vaccines. Vaccine. (2021) 15(39:16):2214–23. 10.1016/j.vaccine.2021.01.060 33658126

[28] LaMontagne DS Manangazira P Marembo J Chigodo C Zvamashakwe C Tshuma E HPV Vaccination coverage in three districts in Zimbabwe following national introduction of 0,12 month schedule among 10 to 14 old girls. Vaccine. (2022) 40(1):58–66. 10.1016/j.vaccine.2021.07.012 34275674

[29] Brown DR Joura EA Yen GP Kothari S Luxembourg A Saah A Systematic literature review of cross-protective effect of HPV vaccines based on data from randomized clinical trials and real-world evidence. Vaccine. (2012) 39(16):2224–36. 10.1016/j.vaccine.2020.11.076 33744051

[30] Carlton JG Marembo J Manangazira P Rupfutse M Sherley A Makwabarara E Nationwide introduction of HPV vaccine in Zimbabwe 2018–2019: experiences with multiple cohort vaccination delivery. PLOS Glob Public Health. (2022) 2(4):e0000101. 10.1371/journal.pgph.0000101 36962162 PMC10021852

[31] Kouamou V Chin'ombe N Matimba A Kadzatsa W Nyandoro G Musarurwa C. P53 codon 72 polymorphism and the risk of cervical cancer in Zimbabwean women. Int J Trop Dis Health. (2016) 15(3):1–6. 10.9734/IJTDH/2016/24676

[32] Pegoraro R Moodley J Naiker S Lanning P Rom L. The p53 codon 72 polymorphism in black South African women and the risk of cervical cancer. BJOG. (2000) 107(9):1164–5. 10.1111/j.1471-0528.2000.tb11118.x 11002963

[33] Assoumou SZ Boumba AL Ndjoyi-Mbiguino A Khattabi A Ennaji MM. The preliminary study of P53 codon 72 polymorphism and risk of cervical carcinoma in gabobese women. Med Oncol. (2015) 32:281–7. 10.1007/s12032-014-0281-4 25502079

[34] Mostaid MS Mumu SB Haque MA Sharmin S Jamiruddin MR Rahman SGM Elevated serum expression of p53 and association codon 72 polymorphisms with risk of cervical cancer in Bangladeshi women. PLoS One. (2021) 16(12):e0261984. 10.1371/journal.pone.0261984 34962972 PMC8714093

[35] Jemia ZB Fehri E Ardhaoui M Jaballah-Gabteni A Lassili T Essafi-Benkhadir K The TP53 rs 1042522 polymorphism and its association with precancerous cervical lesions progression among Tunisian women. Diagn Microbiol Infect Dis. (2025) 113(3):116981. 10.1016/j.diagmicrobio.2025.116981 40609119

[36] dbSNP. Accessible online at: Available online at: https://www.ncbi.nlm.nih.gov/snp/ (Accessed March 11, 2026).

[37] Storey A Thomas M Kalita A Harwood C Gardiol D Mantovani F Role of a p53 polymorphism in the development of human papillomavirus-associated cancer. Nature. (1998) 393:229–34. 9607760 10.1038/30400

[38] Koshiol J Hildesheim A Gonzalez P Bratti MC Porras C Schiffman M Common genetic variation in TP53 and risk of human papillomavirus persistence and progression to CIN3/cancer revisited. Cancer Epidemiol Biomarkers Prev. (2009) 18(5):1631–7. 19423538 10.1158/1055-9965.EPI-08-0830 PMC2764239

[39] Kuguyo O Tsikai N Thomford NE Magwali T Madziyire MG Nhachi CF Genetic susceptibility for cervical cancer in African populations: what are the host genetic drivers? OMICS. (2018) 22(7):468–83. 10.1089/omi.2018.0075 30004844

[40] Siddique MM Balram C Fiszer-Maliszewska L Aggarwal A Tan A Tan P Evidence for selective expression of the p53 codon 72 polymorphs: implications in cancer development. Cancer Epidemiol Biomarkers. (2005) 14(9):2245–52. 10.1158/1055-9965.EPI-05-0153 16172238

[41] Jafrin S Aziz MA Anonna SN Akter T Naznin NE Reza S Association of TP53 Codon 72 arg > pro polymorphism with breast and lung cancer risk in the South Asian population: a meta-analysis. Asian Pac J Cancer Prev. (2020) 21(6):1511–9. 10.31557/APJCP.2020.21.6.1511 32592343 PMC7568897

[42] Jennis M Kung CP Basu S Budina-Kolomets A Let JI Khaku S An African-specific polymorphism in the TP53 gene impairs p53 tumor suppressor function in a mouse model. Genes Dev. (2016) 30(8):918–30. 10.1101/gad.275891.115 27034505 PMC4840298

[43] Murphy ME Liu S Yao S Huo D Liu Q Dolfi SC A functionally significant SNP in TP53 and breast cancer risk in African- American women. NPJ Breast Cancer. (2017) 3:5. 28649645 10.1038/s41523-017-0007-9 PMC5445618

[44] Barnoud T Parris JLD Murphy ME. Common genetic variants in the TP53 pathway and their impact on cancer. J Mol Cell Biol. (2019) 11(7):578–85. 10.1093/jmcb/mjz052 31152665 PMC6736421

[45] Bautista J Altamirano-Colina A Lopez-Cortes A. The vaginal microbiome in HPV persistence and cervical cancer progression. Front Cell Infect Microbiol. (2025) 15:1634251. 10.3389/fcimb.2025.1634251 41127671 PMC12537727

