# Supplementary figures and images for: TP53 codon 47 and 72 polymorphisms show no association with HPV in Zimbabwean women living with HIV and histologically confirmed cervical and vulvar disease
Source: Front Reprod Health. 2026 Apr 20;8:1808566. doi: 10.3389/frph.2026.1808566 (PMC13136159; doi:10.3389/frph.2026.1808566)

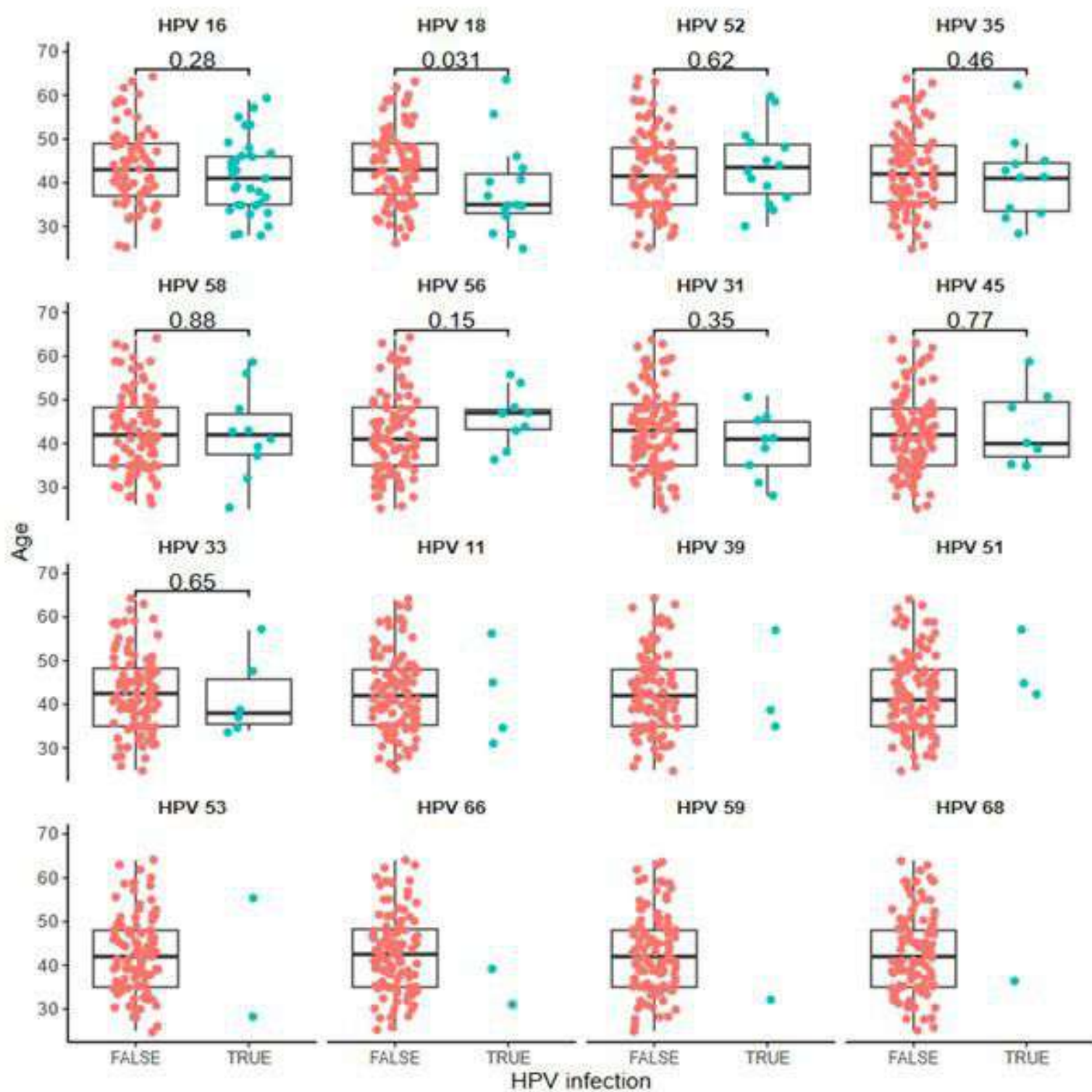

Supplement: Supplementary file 1 [file Datasheet1.pdf]
